# Disease Biomarkers in the Precision Medicine Era: A Comprehensive Multi-Omics Analysis

**DOI:** 10.3390/biomedicines13092218

**Published:** 2025-09-10

**Authors:** Po-Hung Chen, Su-Boon Yong, Chin-Yuan Yii, Chia-Jung Li

**Affiliations:** 1 Department of Emergency Medicine, Kaohsiung Municipal Min-Sheng Hospital, Kaohsiung 802, Taiwan; 2 Department of Allergy and Immunology, China Medical University Children’s Hospital, Taichung 404, Taiwan; 3 Research Center for Allergy, Immunology, and Microbiome (A.I.M.), China Medical University Hospital, Taichung 404, Taiwan; 4 Department of Internal Medicine, Landseed International Hospital, Taoyuan 324, Taiwan; 5 Department of Obstetrics and Gynecology, Kaohsiung Veterans General Hospital, Kaohsiung 813, Taiwan; 6 Institute of BioPharmaceutical Sciences, National Sun Yat-Sen University, Kaohsiung 804, Taiwan

The advent of precision medicine has transformed the landscape of disease biomarker discovery, driven by the integration of genomics, transcriptomics, proteomics, metabolomics, and microbiome profiling. Multi-omics technologies enable the elucidation of disease heterogeneity, the identification of predictive markers, and the development of tailored therapeutic strategies. This Special Issue, “Disease Biomarkers in the Precision Medicine Era: A Comprehensive Multi-Omics Analysis”, highlights five original contributions that collectively advance our understanding of disease pathophysiology and clinical translation.

Bonet et al. profiled the miRNA–mRNA interactome in arrhythmogenic cardiomyopathy, identifying 697 differentially expressed genes and eight key miRNAs, with functional enrichment implicating mitochondrial dysfunction, apoptosis, oxidative stress, and inflammation. Importantly, eleven negatively correlated miRNA–mRNA pairs were highlighted as potential drivers of cardiomyopathy progression [1]. Wang et al. explored the therapeutic potential of resistant starch-encapsulated probiotics (RS-Pro) in colorectal cancer. In a murine 5-fluorouracil (5-FU) model, RS-Pro supplementation inhibited tumor growth, alleviated cachexia, reduced NF-κB signaling, and restored microbial diversity, offering a promising adjunctive approach to chemotherapy [2]. Hsieh et al. investigated cytokines as predictive biomarkers of biologic therapy in psoriasis. They demonstrated that the serum levels of IFN-γ, IL-17A, IL-4, and IL-13 could stratify patients by treatment responsiveness, providing valuable insights into precision immunotherapy for dermatological diseases [3]. Ke et al. examined the role of the immune microenvironment in shaping tumor antigenicity. Tumors established in immunodeficient hosts exhibited a reprogramming of oncogenic signaling pathways, leading to heightened antigenicity. These findings underscore the complex interplay between host immunity and tumor biology, with implications for immuno-oncology biomarker development [4]. Finally, this Special Issue features a comprehensive review addressing the application of omics analyses in pediatric B-cell acute lymphoblastic leukemia (ALL). The authors summarize advances in genomics, transcriptomics, and proteomics, emphasizing how integrated approaches reveal disease heterogeneity, treatment resistance, and potential therapeutic targets in pediatric leukemia [5].

Despite these important advances, several challenges remain before multi-omics biomarkers can be seamlessly translated into clinical applications. One major limitation lies in the complexity of integrating data from different omics layers. Although each platform provides valuable information, the construction of unified frameworks that capture interactions across the genome, transcriptome, proteome, metabolome, and microbiome is still in its infancy. Another concern is clinical validation, as many promising biomarkers have thus far only been tested in preclinical models or relatively small patient cohorts. Without large-scale, multi-center studies, it remains difficult to ensure reproducibility and generalizability across diverse populations. Moreover, most investigations provide only static snapshots of disease states, whereas longitudinal profiling is needed to capture the dynamic trajectories of biomarker expression during disease progression and treatment. Finally, a holistic appreciation of the interplay between host immunity, tumor biology, and the microbiome is still lacking, even though accumulating evidence suggests that these interconnected systems jointly shape disease pathophysiology and therapeutic outcomes. Addressing these challenges will require not only technological and analytical innovation but also interdisciplinary collaboration between clinicians, data scientists, and basic researchers.

The five contributions in this Special Issue exemplify the breadth and promise of multi-omics approaches in advancing precision medicine. By addressing diverse disease contexts ranging from cardiovascular pathology and cancer cachexia to immune modulation and pediatric oncology, these studies collectively highlight the central role of integrative biomarkers in guiding diagnosis, prognosis, and therapy. We sincerely thank all authors, reviewers, and the editorial team for their dedication, and we hope that the findings presented here will inspire continued innovation and collaboration in this exciting field.
